# The Progress of Dentistry

**Published:** 1893-11

**Authors:** 


ARTICLE VI.

### THE PROGRESS OF DENTISTRY.

Baltimore is the cradle of dentistry. Few outside the regular practitioners are aware that here was instituted the first college of dentistry, and that in it was first conferred the degree of D. D. S.—Doctor of Dental Surgery—without which few of the men of the present time deem themselves competent to practice the profession.

In 1839 the Baltimore College of Dental Surgery was organized by Dr. Chapin A. Harris, one of the most eminent practitioners of that day, and for many years the institution, which became a permanency through his efforts, was the only college of the kind in the world.

The art of dentistry is not a new one. It can be traced back to the time of the pyramids, the days when Greece was pre-eminent in everything that pertained to culture and refinement, the hour when Pompeii was inundated by the all-destroying stream of liquid fire, for in the mouths of bodies exhumed from the pile of ashes that was once a prosperous and beautiful city are found teeth which have been filled. Also the instruments of the doctors of that time have been uncovered. The forceps, tweezers, mirrors and the like are in some respects so similar to those of to-day that there can be no mistake as to their usage. Although dentistry existed so far back as the time of Hypocrates, 450 B. C., and probably centuries before up to a short while ago the profession had made comparatively little progress, but within the present century its strides have been remarkable.

Methods, appliances, anæsthetics, totally unknown 30 or 40 years ago, are now in the hands of every dentist who makes any pretension whatever to the practice of his profession.

It is curious that of all nations the people of these

United States have the worst teeth, and this is ascribed to the fact that they are made up of such a conglomerable mixture of different nationalities. A child inherits the teeth of the father, and the jaw of the mother, so that when the two, as very often is the case, are of different races, the teeth of the child are irregular and uneven. Perhaps there is something in the pithy remark of one dentist that "the Father of His Country had false teeth, and all of his children seem inclined to follow his example." However this may be, it is said that the possession of imperfect teeth is a sure sign of civilization, and that it is only among the savage or half savage tribes that the teeth are found in an absolutely perfect state.

American dentists the world over are regarded as the highest representatives of their calling—not due to the fact that there is so much more practice in this country than any other, but because American genius, inventive ability and other attributes assist them to the position they occupy. The proper care of the teeth is essential to the possession of good health, for without good teeth with which to masticate food digestion is not properly performed and some disease of the alimentary canal follows, which is calculated to make life miserable.—*Baltimore Herald.*

